# Immune checkpoint inhibitor–induced toxic epidermal necrolysis responding to repeated dosing of adjuvant therapy with etanercept

**DOI:** 10.1016/j.jdcr.2025.06.040

**Published:** 2025-07-14

**Authors:** Lauren C. LaMonica, Reid Oldenburg, Robert A. Dorschner, Brian Hinds, Jeremy A. Schneider

**Affiliations:** a University of Michigan Medical School, Ann Arbor, Michigan; b Department of Dermatology, University of California San Diego, San Diego, California

**Keywords:** etanercept, immune checkpoint inhibitor therapy, nivolumab, Stevens-Johnson syndrome, toxic epidermal necrolysis, tumor necrosis factor inhibitors

## Introduction

As treatment with immune checkpoint inhibitors (ICIs) becomes increasingly common, the spectrum of immune-related adverse events (irAEs) has widened.^1^ Although rare, severe cutaneous irAEs, including Stevens-Johnson syndrome (SJS)/toxic epidermal necrolysis (TEN), are associated with high mortality. Unique clinical features of SJS/TEN in patients treated with ICIs include gradual onset, with a mean of 11.3 weeks after ICI initiation, and a prodromal morbilliform or papulosquamous eruption preceding epidermal necrosis.^2^ Importantly, relapse occurs if immunosuppression is tapered too early,^2^ suggesting ICI-induced irAEs are more persistent and progressive. Recently, the benefit of combinatorial treatment with systemic corticosteroids and tumor necrosis factor inhibitors (TNFi) has been reported.^3^, ^4^, ^5^ In the largest prospective study, combination corticosteroids and 50 mg etanercept dosed twice weekly until disease stabilization improved outcomes in patients with SJS. This study also showed the benefit of a single dose of infliximab at 5 mg/kg combined with corticosteroids and intravenous immunoglobulin (IVIG) in TEN.^6^ Yet, data in patients with TEN and a SCORTEN (SCORe of Toxic Epidermal Necrolysis) >3 are lacking. Here, we report a patient with ICI-associated TEN who responded to systemic corticosteroids and etanercept, supporting the use of TNF inhibition in patients with severe disease, including those with contraindications to IVIG.

## Case description

**Fig 1:**
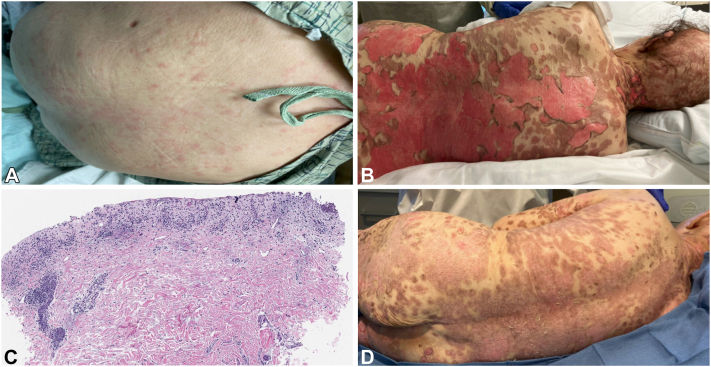
Clinical and histopathologic findings. **A,** Numerous erythematous macules and papules involving the trunk (depicted), thighs, and scalp on hospital day 2. **B,** Erythematous and dusky, diffuse flaccid bullae with desquamation of the trunk on hospital day 10 after administration of the second dose of 50 mg etanercept. **C,** Skin biopsy specimen of the right abdomen demonstrates complete loss of the epidermis and superficial mixed inflammation with an occasional eosinophil with areas of denuded and necrotic epithelium (hematoxylin and eosin; original magnification: ×40). **D,** Halted progression of the rash with interval improvement in desquamation and pinpoint bleeding on hospital day 23 after the sixth dose of etanercept.

A 78-year-old woman diagnosed with stage IVA squamous cell carcinoma of the tongue was treated with 3 cycles of carboplatin, paclitaxel, and pembrolizumab, with subsequent transition to nivolumab and cetuximab (dosed every 2 weeks) 2 months later due to nonresponse. Three days after treatment with nivolumab and cetuximab, she developed a morbilliform eruption. Six days later, she was hospitalized for fevers, painful stomatitis, and persistent rash. Physical examination demonstrated diffuse erythematous macules and patches with overlying bullae, erosions, and oral ulcers (Fig 1, *A*-*C*). Vital signs were normal. Laboratory evaluation was notable for thrombocytosis and elevated transaminases. Indirect immunofluorescence, direct immunofluorescence, and enzyme-linked immunosorbent assay studies did not implicate immunobullous disease. Skin biopsy revealed full-thickness necrotic epithelium with superficial mixed inflammation, including occasional eosinophils (Fig 1, *D*).

Nivolumab and cetuximab were suspected medication triggers, and both of these agents were discontinued during hospitalization. Treatment was initiated with 1 mg/kg methylprednisolone on hospitalization day 2; however, her rash progressed with dusky, diffuse flaccid bullae, mucositis and vulvovaginal involvement, desquamation of 70% to 80% body surface area (BSA), and a SCORTEN of 4 predicting 58.3% mortality (age ≥40 years, malignancy, BSA detached ≥10%, and serum urea ≥10 mmol/L). Based on her clinical progression and advanced malignancy, a risk-benefit discussion of adjuvant therapy was conducted with her interdisciplinary team, and 0.8 mg/kg (50 mg) of etanercept every 4 days (Table I) was initiated on hospitalization day 5. After 2 doses, her rash halted in progression without new erythematous papules or bullae formation, her pain was reduced, and her skin began to heal. Adjuvant etanercept was continued for a total of 8 doses based on persistently elevated inflammatory markers. Unfortunately, after discharge, a computed tomography scan of her neck demonstrated local disease progression, and she was treated with palliative radiotherapy and capecitabine but succumbed to malignancy complications unrelated to TEN 4 months later.

**Table I:** Repeated dosing of adjuvant therapy with etanercept based on inflammatory markers and end-organ damage

Hospital day	Etanercept dose	Corticosteroid dose Intravenous (IV) or Oral (PO)	Inflammatory and end-organ damage markers			
			C-reactive protein (CRP) (Normal: <0.5 mg/dl)	Aspartate Aminotransferase (AST) (Normal: 0-32 U/L)	Alkaline phosphatase (Normal: 40-130 U/L)	Platelet count (Normal: 140-370 1000/mm^3^)
5	1	IV methylprednisolone 1 mg/kg (50 mg)	--	**36 ↑**	**375 ↑**	**780 ↑**
8	2	IV methylprednisolone 1 mg/kg (50 mg)	**0.89 ↑**	27	**277 ↑**	**757 ↑**
12	3	IV methylprednisolone 1 mg/kg (50 mg)	**2.23 ↑**	**51 ↑**	**259 ↑**	**709 ↑**
16	4	IV methylprednisolone 1 mg/kg (50 mg)	**9.27 ↑**	**65 ↑**	**379 ↑**	**533 ↑**
20	5	IV methylprednisolone 1 mg/kg (50 mg)	**5.75 ↑**	20	**280 ↑**	**379 ↑**
24	6	IV methylprednisolone 40 mg	**1.02 ↑**	16	**222 ↑**	**479 ↑**
28	7	IV methylprednisolone 40 mg	0.43	11	**186 ↑**	**485 ↑**
32	8	PO prednisone 35 mg	<0.30	12	**133 ↑**	**419 ↑**

Values exceeding normal range are bolded.

## Discussion

Cutaneous irAEs occur in 34% to 45% of patients on ICI therapy.^7^ The higher incidence of a TEN phenotype indicates that checkpoint inhibition may enhance immune stimulation.^2^ Due to the long half-life of ICIs and time required to reach steady state, cases may have a severe and delayed onset.^1^ Two theories of ICI-associated SJS/TEN have been proposed: the first suggests that ICIs reduce immune tolerance and induce sensitivity to subsequent drugs^1^; the second suggests direct ICI-induced cytotoxicity leading to T-cell targeting of keratinocytes and subsequent apoptosis.^1^^,^^8^ Yet, controversy regarding whether ICI is a distinct entity from SJS/TEN or on the spectrum of SJS/TEN and a true human leukocyte antigen)-mediated Class IV hypersensitivity persists.^2^ Previously, studies have found that among patients with a BSA ≥30%, programmed cell death protein (PD)-1 inhibitors were the most commonly implicated class of ICIs (83%), followed by PD-1/cytotoxic T-lymphocyte-associated protein-4 inhibitors (10.2%) and programmed cell death ligand 1 (4.1%).^2^^,^^9^ While establishing drug association is challenging, our patient was initiated on pembrolizumab, a programmed cell death ligand 1 inhibitor, followed by nivolumab, a PD-1 inhibitor, which may have contributed to direct ICI-induced TEN.

The persistent and refractory nature of ICI-associated SJS/TEN raises important treatment considerations. Corticosteroids remain a common monotherapy for SJS/TEN, and while treatment slows disease progression, corticosteroids have not consistently improved outcomes including mortality or time to complete skin re-epithelization.^3^ The benefit of TNF-alpha blockade has been demonstrated,^5^ with combined systemic corticosteroids and 25 mg of etanercept decreasing mortality, skin healing time, and adverse events related to corticosteroids,^3^, ^4^, ^5^ and with a second etanercept dose administered if skin lesions were not improved.^4^ However, studies have administered etanercept in less severe cases (median SCORTEN ≤3).^6^ Additionally, the only prospective study treating TEN patients with corticosteroids and TNFi (infliximab) also administered IVIG, which may be contraindicated in some critically ill patients.

A gap remains in understanding the effectiveness of TNF-alpha inhibition in treating patients with TEN and more severe disease, highlighting the importance of this case. In our patient (SCORTEN of 4), the decision for repeated etanercept dosing was based on persistently elevated inflammatory markers and hepatitis (Table I); of note, markers of end-organ damage continued to rise until the fourth dose, possibly related to a prolonged acute stage duration for which interleukin-6 has been positively correlated.^5^ While TNF-alpha inhibition must be weighed against immunosuppression risk, there is no evidence of TNF blockade increasing the risk of infection in the acute phase, and our case supports the potential role for prolonged TNF-alpha inhibition given persistent inflammation in ICI-induced cutaneous irAEs.

In our patient, TNF inhibition with etanercept was able to reduce ongoing cutaneous pro-inflammatory cytokine signaling and likely granulysin-mediated keratinocyte cell death, potentially independent of a Class IV hypersensitivity or PD-1-mediated initiation of the TEN spectrum. Comparisons of etanercept and corticosteroid combination therapy, as well as etanercept monotherapy or TNF therapy in combination with other disease-modifying agents, including cyclosporine or IVIG, remain an ongoing area of exploration.^10^ This case supports etanercept bi-weekly dosing for patients with TEN and provides a foundation for future prospective TNF blockade studies in this critically ill population.

## Conflicts of interest

None disclosed.
